# Wearable Contactless Respiration Sensor Based on Multi-Material Fibers Integrated into Textile

**DOI:** 10.3390/s17051050

**Published:** 2017-05-06

**Authors:** Philippe Guay, Stepan Gorgutsa, Sophie LaRochelle, Younes Messaddeq

**Affiliations:** 1 Center for Optics, Photonics and Lasers (COPL), Department of Physics, Université Laval, Québec, QC G1V 0A6, Canada; philippe.guay.4@ulaval.ca (P.G.); sgorgutsa@gmail.com (S.G.); 2 Department of Electrical and Computer Engineering, Université Laval, Québec, QC G1V 0A6, Canada; Sophie.Larochelle@gel.ulaval.ca

**Keywords:** multi-material fibers, textile biosensor, textile respiration sensor, textile RF (Radio Frequency) communications

## Abstract

In this paper, we report on a novel sensor for the contactless monitoring of the respiration rate, made from multi-material fibers arranged in the form of spiral antenna (2.45 GHz central frequency). High flexibility of the used composite metal-glass-polymer fibers permits their integration into a cotton t-shirt without compromising comfort or restricting movement of the user. At the same time, change of the antenna geometry, due to the chest expansion and the displacement of the air volume in the lungs, is found to cause a significant shift of the antenna operational frequency, thus allowing respiration detection. In contrast with many current solutions, respiration is detected without attachment of the electrodes of any kind to the user’s body, neither direct contact of the fiber with the skin is required. Respiration patterns for two male volunteers were recorded with the help of a sensor prototype integrated into standard cotton t-shirt in sitting, standing, and lying scenarios. The typical measured frequency shift for the deep and shallow breathing was found to be in the range 120–200 MHz and 10–15 MHz, respectively. The same spiral fiber antenna is also shown to be suitable for short-range wireless communication, thus allowing respiration data transmission, for example, via the Bluetooth protocol, to mobile handheld devices.

## 1. Introduction

Monitoring vital signals and various activity types using ‘wearable’ electronic devices has become an increasingly attractive field of research, especially in the recent years, appealing to the health and overall well-being concerns of the general public. Consequently, many solutions have surfaced over the last few years, including numerous ‘smart’ watches and fitness trackers. Some of them are aimed at health monitoring, while others focus on recreational usage. 

Numerous studies [1,2,3] show that routine monitoring of basic vital parameters, such as blood pressure, heart rate, body temperature, and respiration rate are extremely useful to expose various medical dysfunctions. While in the present day there are multiple solutions capable, for example, of continuous heart rate monitoring, only a very few [4,5] can also provide respiration rate information. This information, however, is vital for prevention of many respiration disorders. One could think of asthma, pneumonia, chronic obstructive pulmonary disease (COPD), and sleep apnea, as diseases that benefit from the supervision of the breathing rate for improved diagnostics.

Modern techniques used to monitor the respiration rate include pneumography [6], which uses impedance change to monitor the chest movement with the use of electrodes attached to the body, or standard pulse oximetry [7], which allows measurement of the respiration rate through the absorption of infrared light and requires a probe to be linked to the patient’s finger. The respiration rate can also be derived from the measurements given by an electrocardiogram (ECG) [8], which also requires the installation of electrodes on the patient’s body. In the domain of remote biosensing, impulse radio (IR) UWB (ultra-wideband) wireless systems [9] have attracted a lot of interest. These systems rely on the transmission and reception of sub-nanosecond RF (Radio Frequency) pulses for movement detection, including patient’s chest movement which, in turn, allows heartbeat and respiration rate measurements. Such systems do not require any electrodes to be attached to the patient’s body. However, they require complex measurement equipment and signal analysis and, most importantly, restrict the patient to remain in a certain operating zone, i.e., lying in bed. 

On the other hand, in the past decades textiles have become a popular platform for integration of various sensors providing active functionalities to the previously passive textiles. As such, gloves have been developed to detect hand posture [10] and gesture language recognition; pants, to monitor lower body movement [11]; and several shirts, to record ECG [12], electromyography (EMG) [13], electroencephalography (EEG) [14], and breathing rate [5]. Recently there have been attempts to adopt the above-mentioned IR UWB technology into smart textiles [15]. However, these solutions still rely on patch antenna designs, made of conductive threads or fabrics and, thus, according to antenna theory [16], require rather thick (6–7 mm) substrates. Thus, with the development of the smart textiles domain came the realization that conventional microelectronic devices do not satisfy the user-comfort requirements for many practical applications and, hence, emerged the need to develop solutions integrated into threads and fibers composing the textiles. Proposed solutions involve conductive yarn [17], optical fibers [4], conductive polymer [18] or multi-material fibers [19,20], that can be used as sensors [21], antennas [22], or circuit designs [14] incorporated into textiles to monitor various activities. 

Naturally, many smart textile applications are aiming towards medical monitoring. For example, fiber Bragg grating (FBG)-based sensors [4], have been integrated into a cushion to monitor vibrations due to respiration and heart rate. While FBG sensors demonstrate very high precision and are capable of monitoring ECG and respiration rate simultaneously, their most considerable drawback is, however, similar to the IR UWB method: the necessity to use a laser source and often complicated signal reconstruction procedures. That leads to restricted mobility of the user, which can be tolerated in certain applications, for example, during MRI scanning [4]. 

Alternatively, a great variety of various “patch” monitors for long-term ECG measurements [23] has been already proposed. Including piezoelectric elements integrated into a shirt that can provide the breathing rate information [5], and conductive textile patches [24]. However, such solutions often still require either a control unit located somewhere on the shirt or involve the use of probes or electrodes that should be securely attached to the user’s body and can cause certain discomfort. While in some cases uneasiness can be tolerated, for specific applications user comfort is the top-most priority. In particular, it was shown that around 10% of newborn infants require respiratory assistance [25], facing neonatal respiratory disorders. Monitoring the breathing rate of the newborns plays an important role toward the reduction of the interventions needed to assure their wellbeing and is challenging with many classical methods, such as the cardiopulmonary monitor to which newborns are connected via electrodes. This motivated us to focus on the non-invasive respiration monitoring without using any electrodes/probes attached to the body or bulky control units compromising comfort. 

Respiration is made possible by the diaphragm and the external intercostal muscles. During inhalation, the diaphragm contracts itself and moves downward producing a pressure difference causing air to enter the lungs. The contraction of the intercostal muscles causes the ribs to elevate which results in the expansion of the chest cavity allowing a greater volume of air to enter. Typically, the tidal volume which is the volume inhaled during normal breathing is 7 mL/kg [26]. This amount of air penetrates the lungs causing an expansion of 7.37 cm [27] for men aged 25–34 taking deep breathes. The proposed solution takes advantage of the both mechanisms: the physical chest expansion and the change of the air volume in the lungs. 

In this paper, a prototype garment designed to monitor the breathing rate of an adult is proposed. The prototype is made via the integration of the previously-reported multi-material fibers [21,22] in the form of a spiral antenna designed to radiate at 2.45 GHz. The key feature of such antenna is the central frequency shift exhibited due to the lung volume change and textile stretching under the chest movement. This allows providing respiratory data information to a remote PC in real-time. The prototype antenna was first characterized in terms of return loss (*S*_11_), gain, and radiation pattern to assess its performance for the short-range wireless communications. Next, the textile prototype with an integrated antenna was submitted to stretching tests reproducing the respiration movement with and without a body phantom. Finally, on-body measurements were performed to monitor the breathing rate of the two volunteers.

## 2. Sensor Design 

**Figure 1:**
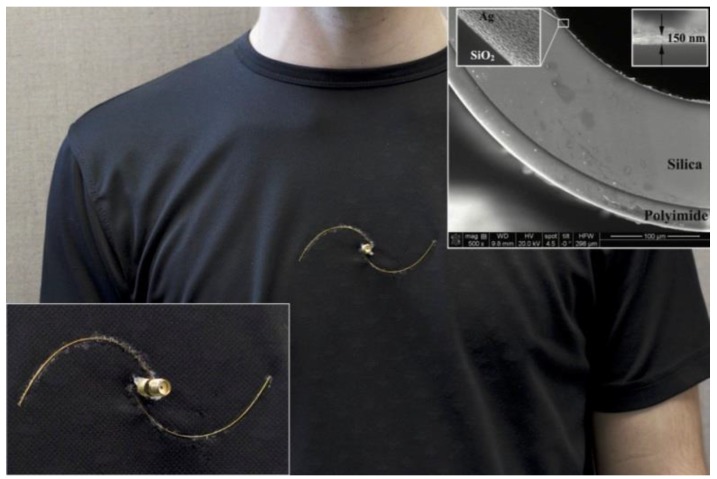
Prototype of the spiral antenna integrated into a cotton shirt, in the inset—SEM images of the multi-material fiber structure.

The spiral antenna is made of multi-material fibers consisting of polyimide-coated hollow-core silica glass capillaries (commercially available from Polymicro Technologies, Phoenix, AZ, USA) in which silver layer was deposited using the liquid state deposition technique based on the Tollen’s reaction [28], as described in our previous publication [21]. The inset of Figure 1a shows the SEM images of the resulting fiber (200 μm inner and 362 μm outer diameter with an 18 μm thick polyimide layer). The inner silver coating layer has a thickness of 150 ± 30 nm, which grants the fibers an electric resistance of 3 ± 1 Ω/cm. The use of multi-material fibers allowed designing an antenna that follows the shape of a half-turn Archimedean spiral. 

As can be seen in Figure 1, position of the spiral arms was adjusted to allow installation of an SMA connector that was used for characterization measurements. Electrical connections were done manually using copper wires (127 μm in diameter). The antennas were integrated into a 20 cm × 10 cm cotton patch (for off-body characterization) and into a cotton t-shirt (for on-body measurements) with cyanoacrylate glue that offers reasonable flexibility. In all experiments, the resonant frequency shift was continuously measured using an HP Agilent 8722ES network analyzer connected to a PC via a GPIB interface. The data acquisition was done with a custom LabVIEW interface.

## 3. Results and Discussion

One of the advantages of the proposed system is that the same fiber antenna could be used to acquire respiration data and to transmit it, for example, to mobile devices. Thus, we first characterize the antenna in terms of its radiation performance (return loss, radiation pattern and gain) in free-space; and then study respiration rate detection mechanisms with the help of human body phantoms and two male volunteers. 

The proposed fiber antenna demonstrates hybrid RF-emissive properties between a multi-turn spiral and a dipole antenna, which can be particularly well seen from the radiation pattern measurements. Hence, the term ‘spiral’ is used for simplicity. 

### 3.1. Antenna Characterization

#### 3.1.1. Return Loss

**Figure 2:**
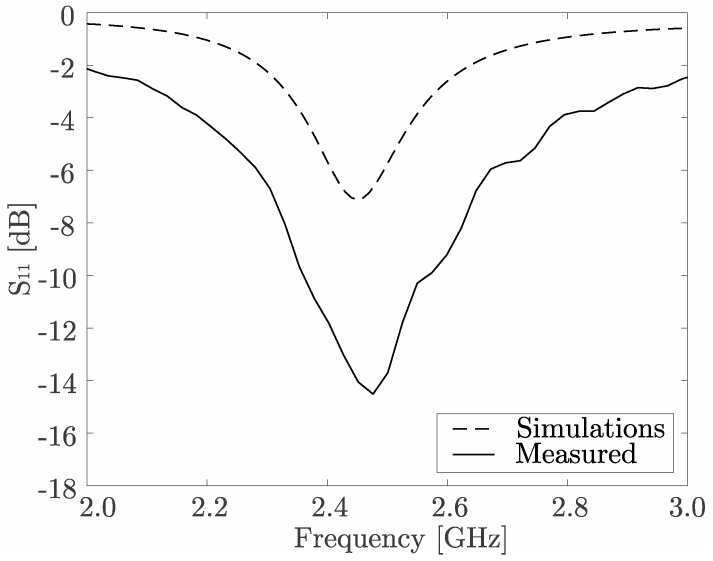
Measured (solid) and simulated (dashed) return loss (*S*_11_) for the spiral antenna.

The concept of scattering (S) parameters, representing input-output relationship between ports (or terminals) of an electrical system, is widely used [29] to characterize frequency responses of the antennas (see Figure 2). The 2.45 [GHz] operating frequency belongs to the ISM (industrial, scientific, and medical) band and was chosen according to the requirements of the medical monitoring applications. Numerical simulations, shown by the dashed line in Figure 2, were done using industry-standard ANSYS HFSS software. The difference between simulations and experimental measurements can probably be attributed to the limitations of the antenna model, since certain assumptions about the material and structure are inevitably made.

#### 3.1.2. Radiation Pattern

**Figure 3:**
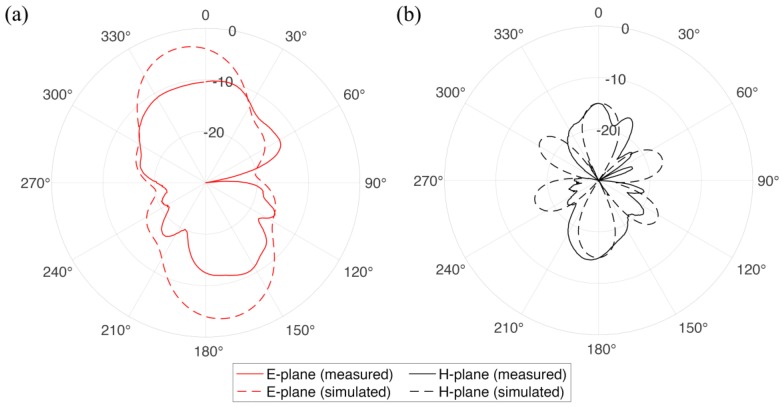
(**a**) E- and (**b**) H- radiation pattern planes of the multi-material fiber spiral antenna operating at 2.4 GHz frequency. Solid and dashed lines correspond to the results of experimental measurements and numerical simulations, respectively.

The radiation pattern refers to the directional dependence of the power emitted (or received) by the antenna and is dependent on many factors: antenna configuration, operating frequency, presence of the other objects, etc. In our case, radiation pattern (see Figure 3a,b) of the textile integrated spiral antenna demonstrates a combination of the classical have-wave dipole radiation pattern and the one of multiple-turns spiral. Experimental measurements were conducted in an anechoic chamber using a known source on the transmission side and textile integrated antenna under study as a far-field receiver. The radiation patterns were measured in an anechoic chamber using wide-band (700 MHz–6 GHz) log-periodic directional antenna (Aaronia HyperLOG-7060 from Aaronia USA, Seneca, SC, USA) and a tunable signal generator at the transmission side with the textile-integrated fiber antenna, fixed on a dielectric holder, acting as the far-field receiver.

#### 3.1.3. Gain

The efficiency of an antenna (*η*) is related to its gain (*G*) and directivity (*D*) as *G* = *ηD* and it is defined as the power radiated relative to the power delivered to the antenna. It can be measured experimentally using the method based on Friis transmission equation [30] described in full details, for example, in one of our previous publications [22]. The line-of-site transmission measurements were performed in an unobstructed lab environment over the distance *R* = 142 cm, with the antennas placed 1 m above the ground an RF absorber to prevent multipath reflections. Both antennas were connected to the network analyzer and gain values determined using the following equation:

(1) $${\left|S_{21}\right|}^{2}=\left(1-{\left|S_{11}\right|}^{2}\right)\left(1-{\left|S_{22}\right|}^{2}\right)G_{T}G_{R}{\left(\frac{c}{4\pi Rf}\right)}^{2}\;$$

where *G_T_* and *G_R_* correspond to the gain and *S*_11_ and *S*_22_ to the return loss of the transmitting and receiving antennas, respectively. In this case, *S*_21_ scattering parameters represents the power transmitted from one antenna to the other at certain frequency, *f*, and *c* is the speed of light. In this case, an antenna with a known gain, *G_T_*, (Aaronia HyperLOG-7060 from Aaronia USA, Seneca, SC, USA) was used on the transmission side. Thus, from Equation (1) it becomes possible to determine gain of the discussed spiral antenna, which equals 3.41 dBi. This value is directly comparable to the 3.45 dBi gain of a common rubber ducky antenna, in our case purchased from Bplus Technology Co. Ltd. (Taipei, Taiwan).

### 3.2. Breathing Detection

**Figure 4:**
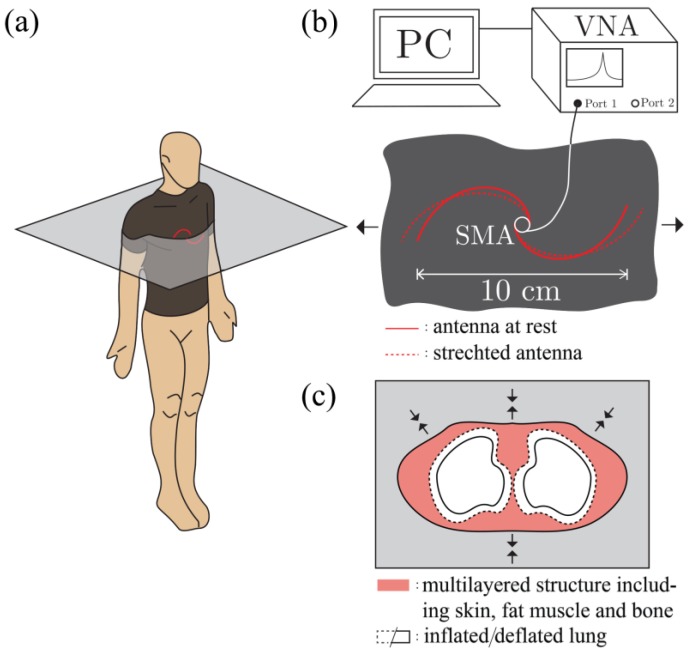
(**a**) Schematic representation of the multi-material fibers spiral antenna integrated into a shirt; (**b**) the spiral antenna configuration change under the stretching load caused by the chest expansion during the breathing; and (**c**) a simplified human torso cross section showing the change of the air volume in the lungs.

As it was mentioned in the introduction, detection of the respiration rate with the proposed textile integrated spiral antenna sensor relies on two mechanisms that are schematically shown in Figure 4. The first mechanism refers to the geometry of the antenna, the second one to the change of dielectric properties of the human torso during breathing. 

The fiber spiral antenna was integrated into a fitted cotton shirt at the mid-chest position (Figure 4a), allowing the chest expansion to slightly stretch the antenna as shown in Figure 4b. It is important to note that such stretching does not cause any elongation or deformation of the metal-glass-polymer fibers themselves, only the radii of the spiral. At the same time, when a person inhales or exhales air from their lungs, the dielectric properties of the whole torso are noticeably changed. For example, the relative permittivity, *ε_r_*, of the inflated lungs at 2.45 GHz equals to 20.51 while the relative permittivity of deflated lungs at the same frequency equals to 48.45 [31]. The performance of the antenna is affected by this variation of the dielectric properties of its surroundings [32], which again results in the central frequency shift. In the next sections, these two mechanisms will be studied with the help of custom mechanical stretching setup and body phantoms. 

#### 3.2.1. Antenna Configuration Change Due to Stretching

##### Off-Body

**Figure 5:**
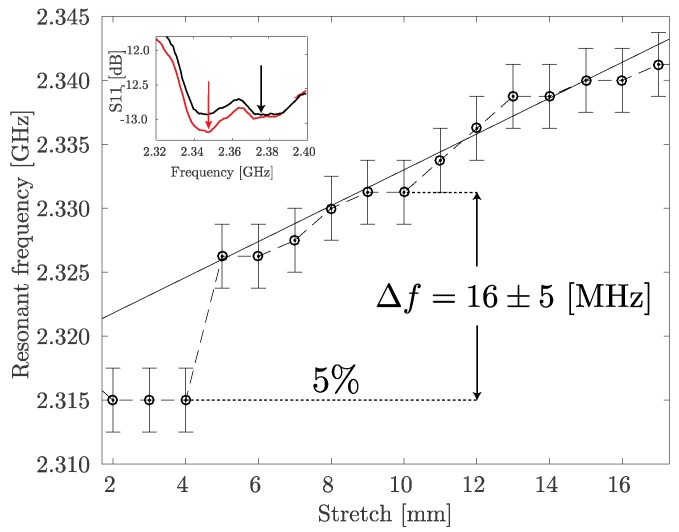
Resonant frequency shift of the textile integrated spiral fiber antenna as a function of the induced stretch in off-body scenario.

In order to assess performance of the multi-material fiber spiral antenna under stretching load it was integrated into 20 cm × 10 cm cotton patch, one side of the patch was fixed by a clamp while the other one was attached to the manual translation stage (1 mm step). The *S*_11_ graphs were measured for every millimeter the textile patch was stretched. Behavior of the resonant frequency of the antenna as a function of the induced stretch is shown in Figure 5. The abrupt change in the 6–8 mm stretch range is explained by the concurrence of the two relative minimums in the *S*_11_ parameter graph as shown in the inset in Figure 5. At the same time, the linear approximation of the 5–16 mm stretch region allows estimation of the respiration sensor sensitivity as 1.4 MHz/mm. It can be seen that 5% stretch (based on the initial 10 cm length of the sample), that corresponds to the expected chest expansion [27], leads to the 16.2 MHz resonant frequency shift. 

##### On body phantom

The close proximity of the human body can significantly alter performance of any antenna [33]. This effect is well known in the field of implantable antennas [34], particularly the downshift of the resonant frequency. Since the sensing mechanism of the proposed spiral fiber antenna also relies on the resonant frequency shift, it becomes crucial to investigate its performance in the proximity of a human body phantom. Therefore, the stretching test described above was repeated in the presence of a 17 cm × 12 cm × 3 cm body phantom at 6 mm distance from the textile sample plane. While the 6 mm distance originates from the physical constraints of the experimental setup, it also seems reasonable for a standard scenario of a normal shirt on the torso of a user. The human body is a very complex, inhomogeneous, layered structure with different tissues having different dielectric properties [31] and the great variety of corresponding models have already been developed [35]. In this case, a body phantom representing only muscle tissue was used. It was fabricated according to the guidelines found in the literature [36], particularly 60% (by weight) of deionized water, 40% of sugar, and 2 g of gelatin powder per 100 mL. 

**Figure 6:**
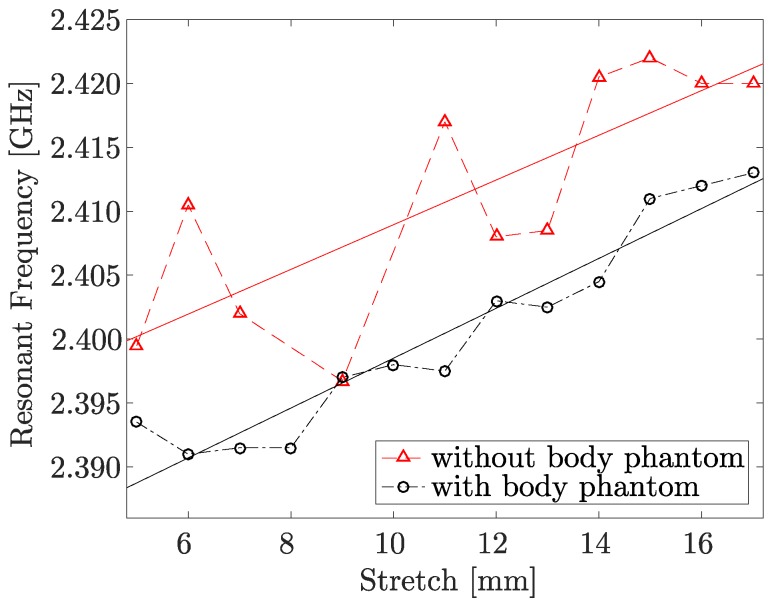
Resonant frequency shift of the textile integrated spiral fiber antenna as a function of the induced stretch with and without the body phantom.

In Figure 6, the resonant frequency shift of the textile integrated spiral fiber antenna as a function of the induced stretch with and without the body phantom is shown. It can be seen that although the absolute frequency value with body phantom is lower, the relative frequency shift is still easily detectable and for the 5% stretch equals to approximately 6 MHz. The results shown in Figure 5 and Figure 6 were obtained with different prototypes (of the same design) and, thus, there is a certain variation in the resonant frequency, which is not crucial since the sensing mechanism depends on its change rather than absolute value.

#### 3.2.2. Respiration Sensing Based on the Torso Volume Change

**Figure 7:**
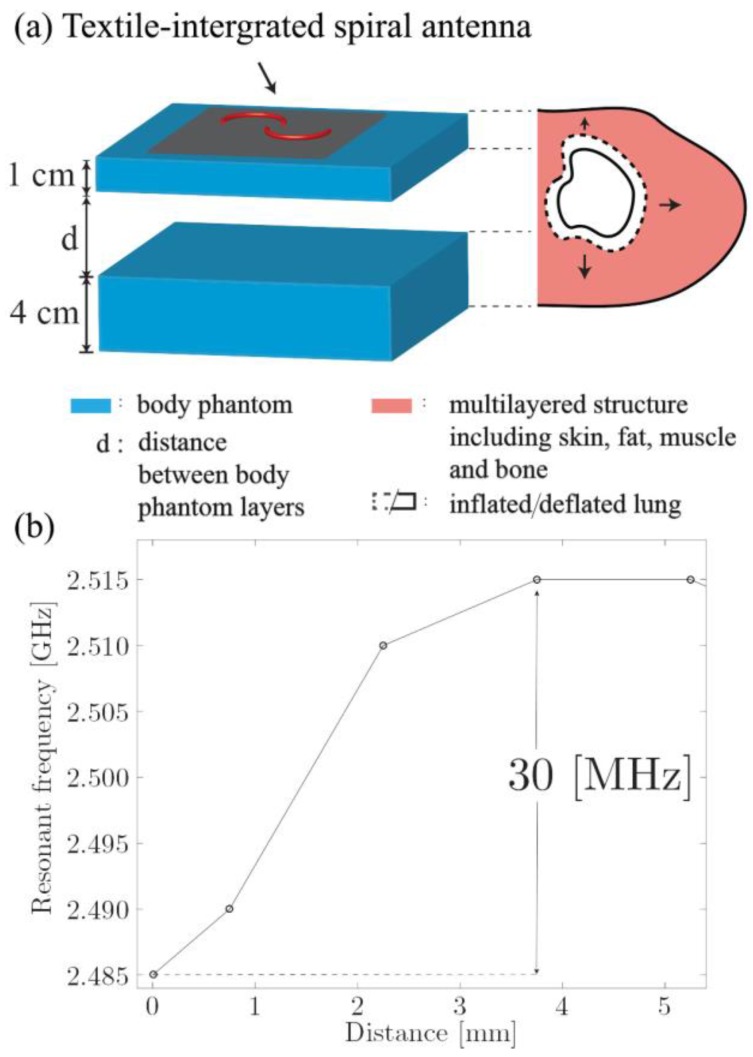
(**a**) Two-layer human body phantom setup to replicate chest movement during breathing; (**b**) the resonant frequency of the integrated multi-material fiber antenna depending on the distance, d, between the phantom layers.

The second mechanism involved into respiration sensing with the proposed spiral antenna is related to the change of the air volume inside the lungs and inner configuration of the thoracic cage. In order to investigate this mechanism another experiment was conducted, this time using the two-layer body phantom as shown in Figure 7. This setup was aimed to reproduce the breathing movement when the back of the subject would remain steady and contraction of the intercostal muscles would force the expansion of the chest. It should be noted that this is of course an oversimplification of the real situation as the setup does not take into account the real structure of the thoracic cage and displacement distance will be different for each user and depend on the position of the antenna on the user’s chest. However, this kind of measurements still can be used to illustrate the discussed effect. 

The shift of the spiral antenna resonant frequency depending on the displacement of the second phantom layer is shown in Figure 7b. It can be seen that, for example, the displacement of 3.6 mm leads to the significant shift of 30 MHz. The same increase in the chest diameter, if considering the lungs as two cylinders with the height of 20.5 cm and a volume of 2262 cm^3^, as reported in [37], will result in the 312 mL volume change. While, tidal volume intakes are often estimated as 7 mL/kg [26], which for a 75 kg body mass yields 525 mL. This is, of course, a very rough estimation, which is only meant to provide a reference point. From one side, the real human body is a much more complex and inhomogeneous structure. From the other, the discussed frequency shift occurs towards the higher frequencies, the same as in the case of pure mechanical stretching (see Figure 6). Therefore, it is reasonable to expect that these two mechanisms combined together in the real-world scenario will result in a greater frequency shift, thus increasing sensitivity of the proposed sensor. 

#### 3.2.3. On-Body Measurements

**Figure 8:**
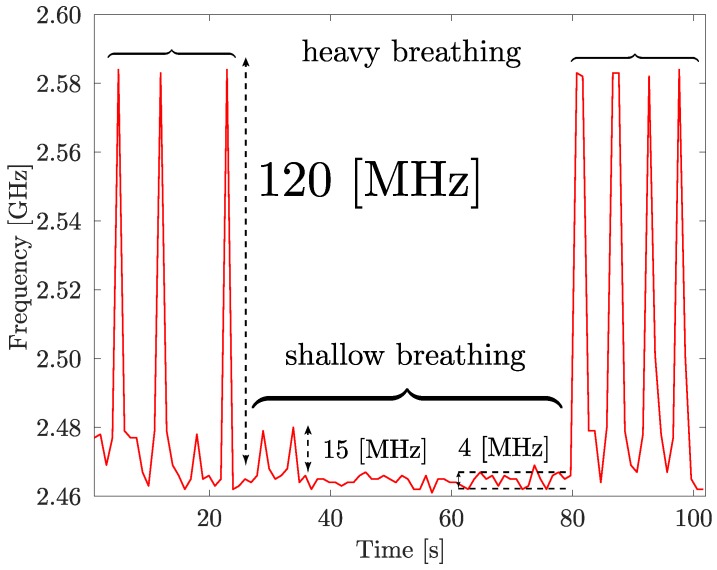
Resonant frequency of the multi-material fiber antenna integrated into textile as a function of time during breathing pattern measurements of an adult male volunteer (standing).

Finally, real-world respiration tests were conducted with the help of two male volunteers. The volunteer whose breathing pattern is presented in Figure 8 was sitting steadily during the recording and could not see the data being recorded. The volunteer was asked to take four deep breaths, followed by one minute of relaxed, shallow breathing, and four more deeps breaths. As before, the resonant frequency shift was continuously measured using a HP Agilent 8722ES network analyzer connected to a PC via a GPIB interface. The acquisition rate was set to provide one measurement per second. 

From Figure 8 it can be seen that the two mechanisms, combined, result in a noticeable frequency shift that allowed the correct detection of the breathing pattern of the volunteer. 

**Figure 9:**
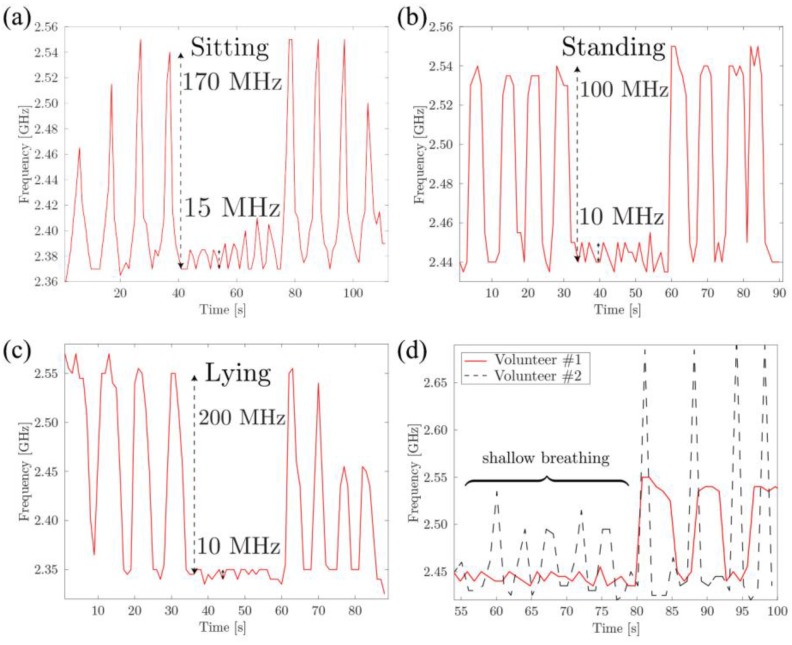
Breathing patterns of an adult male volunteer in (**a**) sitting, (**b**) standing, and (**c**) lying scenarios. (**d**) The comparison of the breathing patterns (standing) for two male volunteers, the time scales are synchronized by the first deep breath for each volunteer.

For deep breathing, frequency shifts as large as 120 MHz were detected, while relaxed, shallow breathing led to smaller, 4–15 MHz, but still detectable, frequency shifts. The measurements were also repeated in sitting and lying positions, as well as with the help of a second male volunteer, as shown in Figure 9a–d, respectively. From the obtained results, several important conclusions can be made. First, the absolute value of the frequency shift is different for different scenarios. For example, shallow breathing peaks (15 MHz) are clearly more pronounced in the sitting position (Figure 9a) and deep breathing peaks are almost twice as large in in the lying position (200 MHz, see Figure 9c) as in the standing (100 MHz) scenario (Figure 9b). This can be explained both by the change in the configuration and position of the sensor and by the change in the user’s chest movement while breathing. Still, the breathing pattern itself (four deep breaths, eight shallow breaths, four more deep breaths) was unmistakably detected in all the test scenarios. Second, by comparing two graphs obtained for two volunteers (Figure 9d) the differences in breathing patterns can be observed (the second volunteer has a larger volume of the lungs and generally takes sharper breaths).

Once again, while the results of these measurements seem to be very promising, they are still at the preliminary stage. The goal of this work is to validate the proposed respiration sensor based on multi-material fibers, integrated into a standard cotton shirt in the spiral antenna arrangement, for the respiration rate detection. Head-to-head comparison with the gold standard of spirometry or pneumography measurements, as well as additional tests, for example, on patients with respiratory problems, requires input from the qualified medical personnel and special equipment, and should become a subject of the future work. The main advantage of the proposed approach consists of high user comfort associated with the traditional garments, as it does not require direct skin contact or attachment of the electrodes of any kind. This becomes particularly important in such cases as monitoring of the respiration rate of the newborn infants. In the experiments reported in this paper the S_11_ graphs and, thus, resonant frequency of the spiral fiber antenna, were continuously measured using a vector network analyzer. However, for mobile applications another detection schemes can be used, for example, based on the measurement of the power transmitted through the narrow band filter over wireless communication network belonging to the ISM band, such as Bluetooth. In the previous work of our group [19] it was demonstrated that multi-material fiber antennas can be effectively used for data transmission over Bluetooth with the help of commercially-available, off-the-shelf miniature transceivers.

## 4. Conclusions

In conclusion, contactless textile integrated respiration sensor suitable for detection, and simultaneous transmission over civilian wireless networks, of the wide range of breathing patterns was demonstrated. The sensor was made using multi-material metal-glass-polymer fibers in the arrangement of a spiral antenna designed to operate at 2.4 GHz frequency. Off- and on-body measurements have shown that the breathing detection is made possible by the two mechanisms: the change of the antenna configuration due to mechanical stretching, and the change of dielectric properties of the human torso during the respiration. Both effects contribute to the shift of the antenna resonant frequency toward higher frequency range. Respiration patterns for two male volunteers were recorded with the help of a sensor prototype integrated into standard cotton t-shirt in sitting, standing and lying scenarios. Typical measured frequency shifts for the deep breathing lies in the range of 120–200 MHz, and 10–15 MHz for shallow breathing. Future work will aim the adaptation of the technology to newborn infants where comfort is a priority.
